# S9-5 From a successful RCT to community implementation: lessons learnt from the REtirement in ACTion (REACT) group-based active ageing intervention targeting older adults with mobility limitations

**DOI:** 10.1093/eurpub/ckad133.047

**Published:** 2023-09-11

**Authors:** Afroditi Stathi, Janet Withall, Colin J Greaves, Janice L Thompson, Max Western, Peter Ladlow, Kenneth R Fox

**Affiliations:** University of Birmingham; University of Birmingham; University of Birmingham; University of Birmingham; University of Bath; UK Ministry of Defence; University of Bristol

## Abstract

**Purpose:**

Mobility limitation in older age reduces quality of life, generates substantial health and social care costs, and increases mortality. The REtirement in ACTion (REACT) trial established that a community-based, active ageing intervention can prevent decline in physical functioning in older adults already at increased risk of mobility limitations documenting significant savings in health and social care costs.

**Project description:**

Recruitment: We recruited 777 older adults (mean age 77.6 yrs (SD 6.8 yrs); 66% female; with reduced lower limb physical functioning (Short Physical Performance Battery [SPPB] mean score 7.37, (SD 1.56) from three sites (Bristol/Bath; Birmingham; Exeter) in England. In terms of ethnicity (95.11% white) and deprivation, the sample was representative of the UK population of people 65+.

**Intervention:**

Participants were randomly assigned to receive three healthy ageing sessions (n = 367) or a 12-month, group-based, multimodal programme including 64x1hr exercise, 43x20 min social and 21x45-min behavioural maintenance sessions (n = 410) delivered by qualified exercise specialists.

**Implementation:**

The intervention was funded and delivered by community organisations including city councils, third sector organisations/charities, leisure industry providers. Currently REACT is implemented as a community programme in Bristol, UK while a strategy for rolling-out nationally is developed.

**Evaluation:**

A multicentre, pragmatic, randomised controlled trial with process and health economic evaluations at baseline, six, 12 and 24 months. At the 24-month follow-up, the SPPB score was significantly greater in the intervention arm than in the control arm. Difference in lower limb function between intervention and control participants was clinically meaningful at both 12 and 24 months. Attrition rates were low (19%). Engagement with the REACT intervention was associated with positive changes in exercise competence, relatedness, enjoyment and perceived well-being benefits.

The health economic analysis indicated substantial quality-of-life and health economic benefits within the 24-month trial window and across a lifetime horizon.

**Conclusions:**

This study adds robust evidence that a relatively low-resource, 1-year multimodal exercise,social and behavioural maintenance intervention helps older adults to improve and retain physical functioning for at least 24 months. There are great opportunities and some challenges for a national and international roll-out of this evidence-based active ageing programme.

